# Weekly 17 alpha-hydroxyprogesterone caproate to prevent preterm birth among women living with HIV: a randomised, double-blind, placebo-controlled trial

**DOI:** 10.1016/S2352-3018(21)00150-8

**Published:** 2021-09-09

**Authors:** Joan T Price, Bellington Vwalika, Bethany L Freeman, Stephen R Cole, Pooja T Saha, Felistas M Mbewe, Winifreda M Phiri, Marc Peterson, Dorothy Muyangwa, Ntazana Sindano, Humphrey Mwape, Megan E Smithmyer, Margaret P Kasaro, Dwight J Rouse, Robert L Goldenberg, Elwyn Chomba, Jeffrey S A Stringer

**Affiliations:** a Department of Obstetrics and Gynecology, University of North Carolina at Chapel Hill, NC, USA; b Biostatistics, University of North Carolina at Chapel Hill, NC, USA; c Epidemiology, University of North Carolina at Chapel Hill, NC, USA; d Departments of Obstetrics and Gynaecology, University of Zambia School of Medicine, Lusaka, Zambia; e Paediatrics, University of Zambia School of Medicine, Lusaka, Zambia; f UNC Global Projects–Zambia, Lusaka, Zambia; g Department of Obstetrics and Gynecology, Brown University School of Medicine, Providence, RI, USA; h Department of Obstetrics and Gynecology, Columbia University, New York, NY, USA

## Abstract

**Background:**

Women with HIV face an increased risk of preterm birth. 17 alpha-hydroxyprogesterone caproate (17P) has been shown in some trials to reduce early delivery among women with a history of spontaneous preterm birth. We investigated whether 17P would reduce this risk among women with HIV.

**Methods:**

We did a randomised, double-blind, placebo-controlled trial in pregnant women with HIV at the University Teaching Hospital and Kamwala District Health Centre in Lusaka, Zambia. Eligible patients were women aged 18 years or older with confirmed HIV-1 infection, viable intrauterine singleton pregnancy at less than 24 weeks of gestation, and were receiving or intending to commence antiretroviral therapy during pregnancy. Exclusion criteria were major uterine or fetal anomaly; planned or in situ cervical cerclage; evidence of threatened miscarriage, preterm labour, or ruptured membranes at screening; medical contraindication to 17P; previous participation in the trial; or history of spontaneous preterm birth. Eligible participants provided written informed consent and were randomly assigned (1:1) to receive 250 mg intramuscular 17P or placebo once per week, starting between 16 and 24 weeks of gestation until delivery, stillbirth, or reaching term (37 weeks). Participants and study staff were masked to assignment, except for pharmacy staff who did random assignment and prepared injections but did not interact with participants. The primary outcome was a composite of delivery before 37 weeks or stillbirth at any gestational age. Patients attended weekly visits for study drug injections and antenatal care. We estimated the absolute and relative difference in risk of the primary outcome and safety events between treatment groups by intention to treat. This trial is registered with ClinicalTrials.gov, NCT03297216, and is complete.

**Findings:**

Between Feb 7, 2018 and Jan 13, 2020, we assessed 1042 women for inclusion into the study. 242 women were excluded after additional assessments, and 800 eligible patients were enrolled and randomly assigned to receive intramuscular 17P (n=399) or placebo (n=401). Baseline characteristics were similar between groups. Adherence to study drug injections was 98% in both groups, no patients were lost to follow-up, and the final post-partum visit was on Aug 6, 2020. 36 (9%) of 399 participants assigned to 17P had preterm birth or stillbirth, compared with 36 (9%) of 401 patients assigned to placebo (risk difference 0·1, 95% CI −3·9 to 4·0; relative risk 1·0, 95% CI 0·6 to 1·6; p=0·98). Intervention-related adverse events were reported by 140 (18%) of 800 participants and occurred in similar proportions in both randomisation groups. No serious adverse events were reported.

**Interpretation:**

Although 17P seems to be safe and acceptable to participants, available data do not support the use of the drug to prevent preterm birth among women whose risk derives solely from HIV infection. The low risk of preterm birth in both randomisation groups warrants further investigation.

**Funding:**

US National Institutes of Health and the Bill and Melinda Gates Foundation.

## Introduction

Women living with HIV face a substantially increased risk of preterm birth,^1^ the most common cause of mortality in neonates and children younger than 5 years worldwide.^2^ In sub-Saharan Africa, where most pregnant women with HIV live, nearly one in five infants are born preterm.^3^ Although expanding access to antiretroviral therapy (ART) in pregnancy has greatly reduced the global burden of paediatric HIV, it does not consistently mitigate the risk of preterm birth or prematurity-related neonatal death.^4^

Interventions to prevent preterm birth have been the subject of investigation for decades, but few have shown efficacy.^5^ Trials have shown vaginal micronised progesterone reduces early delivery among women with a shortened uterine cervix, and intramuscular 17 alpha-hydroxyprogesterone caproate (17P) reduces early delivery among women with a history of spontaneous preterm birth.^6^, ^7^ These drugs continue to be prescribed in the USA and elsewhere, although trials have called their efficacy into question.^8^, ^9^

**Research in context**

**Evidence before this study**

Maternal HIV infection is strongly associated with preterm birth. A 2013 Cochrane Review reported that antenatal 17 alpha-hydroxyprogesterone caproate (17P) reduces the risk of recurrent preterm birth among women who had a previous spontaneous preterm birth of a singleton infant. 17P is often prescribed in North America and parts of Europe for this indication. However, in a nine-country confirmatory trial mandated by the US Food and Drug Administration, 17P was not effective for prevention of recurrent preterm birth. We did a search of the published literature in PubMed and MEDLINE using the terms “HIV”, “preterm”, “prematurity”, and “17-hydroxyprogesterone caproate” or “17-hydroxyprogesterone caproate” or “17P” or “progesterone”. The search was limited to randomised trials published between database inception and May 7, 2016. We found no trials of 17P in pregnant women living with HIV. The search was updated on Dec 30, 2020, and no additional trials were identified.

**Added value of this study**

To our knowledge, ours is the first phase 3 randomised trial of antenatal progesterone supplementation to prevent preterm birth among women living with HIV, and the second largest trial of 17P for any indication. In this study, weekly 17P did not reduce the composite risk of preterm birth or stillbirth in women with confirmed HIV infection and no history of preterm birth. Very high adherence to study product and complete ascertainment of the primary outcome underscore the reliability of these conclusions.

**Implications of all the available evidence**

Although 17P seems to be safe and acceptable to participants, available data do not support the use of the drug to prevent preterm birth among women whose risk derives solely from HIV infection. Whether 17P is effective among women with HIV and previous spontaneous preterm birth remains unknown. As new preventive interventions are introduced, efficacy trials among women with HIV and those living in sub-Saharan Africa are crucial.

HIV infection is associated with systemic and local (vaginal) inflammation,^10^ both of which are common antecedents of spontaneous preterm birth.^11^ Zambian treatment guidelines recommend immediate ART initiation for all people diagnosed with HIV, including pregnant and breastfeeding women.^12^ The current first-line antiretroviral regimen for pregnant women in Zambia is combination tenofovir disoproxil fumarate, emtricitabine, and efavirenz, with second-line therapy being combination zidovudine, emtricitabine, and a boosted protease inhibitor. The risk conferred by HIV in pregnancy seems to occur among nulliparous women and parous women with or without a history of preterm birth.^1^ We hypothesised that the broad anti-inflammatory properties of progesterone could minimise HIV-mediated immune activation and inflammation and thereby reduce the risk of preterm birth related to maternal HIV.

## Methods

### Study design

The Improving Pregnancy Outcomes with Progesterone (IPOP) study is a phase 3, randomised, double-blind, placebo-controlled trial done at the University Teaching Hospital and Kamwala District Health Centre in Lusaka, Zambia. This study protocol^13^ was approved by the University of North Carolina Institutional Review Board (reference 17-1173), the University of Zambia Biomedical Research Ethics Committee (reference 015-06-17), the Zambia Medicines Regulatory Authority (reference CT-070), and the Zambia National Health Research Authority (reference MH/101/23/10/1) before initiation. An external monitor did quarterly visits to trial sites to ensure that the study was done, recorded, and reported in accordance with the protocol, standard operating procedures, International Conference on Harmonization Good Clinical Practice, and US 45 Code of Federal Regulations 46 requirements. This study was designed and implemented following the Consolidated Standards of Reporting Trials Statement and the Standards for Protocol Items: Recommendations for Interventional Trials. Participants were reimbursed for their time and transportation costs.

### Participants

Eligible patients were women aged 18 years or older with confirmed HIV-1 infection, viable intrauterine singleton pregnancy at less than 24 weeks of gestation, were receiving or intending to commence ART in pregnancy, were able to provide written informed consent, intended to remain in Lusaka for the duration of the study, and were willing to adhere to the study visit schedule. We excluded women with known uterine anomaly; planned or in situ cervical cerclage; known major fetal anomaly; indication for planned delivery at less than 37 weeks; evidence of threatened miscarriage, preterm labour, or ruptured membranes; contraindication to 17P as listed in the prescribing information; previous participation in the trial; and any other condition that would make trial participation unsafe or complicate data interpretation. We also excluded women with a previous spontaneous preterm delivery because, at the time of study planning, these women would have been eligible to receive 17P for that indication. Recruitment activity included community sensitisation to encourage interest in the trial and early antenatal care presentation, and antenatal health talks at the recruitment clinics. Study staff identified potential participants attending antenatal care before 24 weeks of gestation. Patients completed written informed consent in English, Nyanja, or Bemba.

### Randomisation and masking

Patients were randomly assigned (1:1) to receive 17P or placebo manufactured to be indistinguishable from the active drug but containing only the excipients castor oil, benzyl benzoate, and benzyl alcohol. A statistician from the University of North Carolina Center for AIDS Research not otherwise associated with the study created the randomisation scheme using random permuted blocks. Participants and study staff were masked to randomisation group, except for pharmacy staff, who did the random assignments using a web-based tool and prepared injections but did not interact with participants. Electronic records of random assignment were kept on this password-protected, web-based tool. Paper documentation of random assignment and allocation of study product were kept in locked filing cabinets within the locked pharmacy, accessible only to unmasked pharmacy staff.

### Procedures

Detailed study procedures have been published previously.^13^ Potentially eligible women received ultrasonography for gestational age determination and, for women between 16 and 24 weeks of gestation, cervical length by transvaginal ultrasound. Study staff then verified antenatal and HIV history data by baseline questionnaire and did a review of medical records, physical examination, and point of care screening procedures. We tested all participants for HIV at the screening visit and confirmed all positive cases with the SD Bioline 3.0 test (SD Biostandard Diagnostics, Gurgaon, India). We drew maternal venous samples to assess HIV plasma viral load (Gene Xpert HIV-1 assay; Cepheid, Sunnyvale, CA, USA) and CD4 cell count (Cytomics FC500 Flow Cytometer; Beckman Coulter Diagnostics, Brea, CA, USA). Eligible participants returned to the research clinic between 16 and 24 gestational weeks for random assignment and initiation of study drug (intramuscular 17P 250 mg [1 mL] once per week) or placebo. Thereafter, participants attended weekly visits for study drug injections and antenatal care. Study drug was discontinued once the participant reached 37 weeks or had the primary outcome. Both 17P and placebo were donated by AMAG Pharmaceuticals (Waltham, MA, USA) who had no other role in the study.

### Outcomes

The primary outcome was a composite of preterm birth (delivery occurring after random assignment and before 37 gestational weeks) or stillbirth (fetal death diagnosed before delivery or delivery of a neonate without signs of life). Secondary outcomes were spontaneous delivery before 37 gestational weeks, delivery before 34 weeks or 28 weeks, infant birthweight below the 10th percentile or below the 3rd percentile for gestational age, mother-to-child HIV transmission, neonatal and perinatal mortality rates, and infant Apgar scores. Safety outcomes were assessed and documented at each participant contact. These outcomes included death (maternal, fetal, or neonatal), drug-related reactions, birthweight less than 2500 g or 1500 g, maternal inflammation or immune activation, alterations of the vaginal microbiome, infant morbidity, and serious adverse events or events that resulted in study discontinuation. Study staff obtained interval information about the maternal and neonatal clinical course through medical record review, direct assessment, and participant self-report at delivery and again at a clinic visit 6 weeks after delivery. Infant HIV infection was assessed by viral load assay (Gene Xpert HIV-1 Qual assay; Cepheid, Sunnyvale, CA, USA) of heel-prick dried blood spot specimens obtained at 6 weeks of life.

### Statistical analysis

We estimated^13^ that 17P would reduce the primary outcome by 38%^6^ (from 24% to 15%) with up to 15% loss to follow-up. We therefore intended to recruit 400 women per group to provide 80% statistical power based on a two-sided Fisher's exact test with significance of 0·05.

All participants were analysed according to their randomised group (ie, intention to treat). For primary and secondary analyses, we estimated the absolute risk, difference in risk, and relative risk (RR) of each outcome, with Wald-type 95% CIs. We planned to use the Kaplan-Meier method to account for right censoring, but this was not required because of complete follow-up. We report the cumulative incidence of the primary outcome by randomised group as a function of time from random assignment; term livebirths remained in the risk set for this analysis and were treated as competing events. Reported p values are from two-sided χ^2^ tests (Gray's test when comparing cumulative incidence curves).

Medication adherence is reported as the proportion of expected study drug injections that were received, with expected doses defined as the number of weeks between random assignment and the first of preterm delivery, stillbirth, or 37 weeks of gestation.

An independent Data Safety and Monitoring Board evaluated the progress of the trial and provided recommendations to study investigators at 6, 9, 18, and 27 months from study initiation. Using the O'Brien-Fleming stopping rule,^14^ statistical significance was to be declared on the 18-month interim test if p<0·0053. In this final analysis, significance was set as p<0·0485. All statistical analyses were done with SAS version 9.4 (Cary, NC, USA). This trial is registered with ClinicalTrials.gov, NCT03297216.

### Role of the funding source

The funders of this study contributed to initial study design but had no role in study implementation, data collection, data analysis, data interpretation, or writing of this report.

## Results

**Figure 1:**
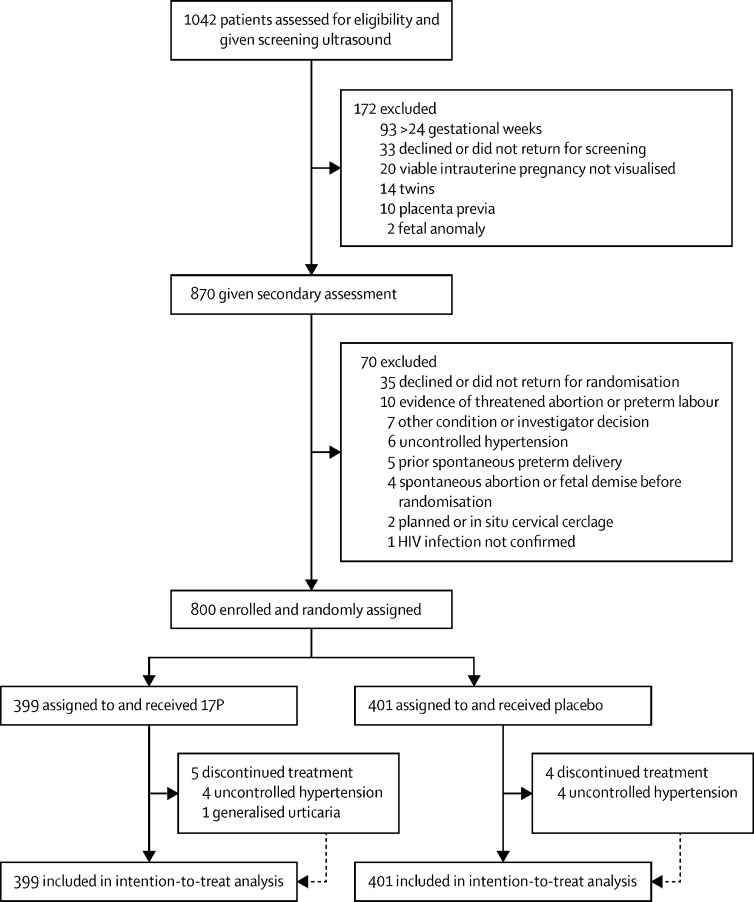
Trial profile 17P=17-alpha hydroxyprogesterone caproate.

Between Feb 7, 2018, and Jan 13, 2020, we identified 1042 women as potentially eligible for the study (figure 1). 800 eligible patients (429 from University Teaching Hospital and 371 from Kamwala District Health Centre) were enrolled and randomly assigned to receive intramuscular 17P (n=399) or placebo (n=401). No patients were lost to follow-up, and the final post-partum visit was on Aug 6, 2020. Baseline characteristics were similar between groups (table 1). Of 594 participants who entered antenatal care with a known HIV diagnosis, 584 (98%) had initiated ART before conception. The remaining 216 participants started ART during pregnancy. 365 (46%) of 800 participants had detectable virus at baseline. In these patients, mean viral load was 1270 copies per mL (IQR 188–21 900). 405 (69%) of 584 women who had started treatment before pregnancy had undetectable viral load at baseline. Median CD4 count among all participants was 568 cells per μL (IQR 367–765), and 50 (6%) had CD4 counts less than 200 cells per μL. Most participants received tenofovir, emtricitabine, and either efavirenz (n=759; 95%) or nevirapine (n=11; 1%). 24 (3%) received a protease inhibitor-containing regimen, and the remaining 1% were on another regimen.

**Table 1:** Baseline characteristics

		**17P (n=399)**	**Placebo (n=401)**
Median age, years		29 (25–33)	30 (25–34)
Body-mass index, kg/m^2^		25·4 (22·7–28·4)	25·3 (22·9–29·0)
	<18·5	6 (2%)	6 (2%)
	18·5–30	321 (80%)	322 (80%)
	>30	72 (18%)	73 (18%)
Parous		323 (81%)	320 (80%)
Previous stillbirth		14 (4%)	8 (3%)
Gestational age at screening, weeks		18·7 (15·6–21·1)	17·9 (15·3–20·7)
Gestational age at random assignment, weeks		19·3 (16·9–21·6)	18·7 (16·7–21·3)
Transvaginal cervical length, cm		4·1 (3·5–4·6)	4·0 (3·6–4·6)
	Short cervix (<2·5 cm)	1 (<1%)	3 (1%)
ART initiation			
	Before pregnancy	295 (74%)	289 (72%)
	During pregnancy	104 (26%)	112 (28%)
Viral load undetectable		218 (55%)	217 (54%)
Viral load among detectable, copies per mL		1470 (188–27 000), n=181	905 (191–20 850), n=184
CD4 count, cells per μL		582 (364–750)	550 (370–778)
	<200	23 (6%)	27 (7%)
Syphilis in pregnancy		60 (15%)	61 (15%)
Bacteriuria*		48 (12%)	46 (11%)
Haemoglobin, g/dL		12·2 (11·1–13·3)	12·1 (11·0–13·1)
	<11	39 (10%)	44 (11%)
Education, years		9 (7–12)	9 (7–12)
	Did not complete primary	138 (35%)	149 (37%)
Marital status			
	Neither married nor cohabiting with partner	60 (15%)	66 (16%)
	Married or cohabiting with partner	339 (85%)	335 (84%)
Alcohol use during pregnancy†		43/398 (11%)	35/401 (9%)
Tobacco use during pregnancy		0	0

Data are n (%), median (IQR), or n/N (%). 17P=17 alpha-hydroxyprogesterone caproate. ART=antiretroviral therapy.

* Defined as 3 or more leukocyte esterase or nitrites on urine dipstick.

† One woman in 17P group did not answer this question.

Of 14 247 expected study injections, 257 (2%) were missed because of non-adherence and 33 (<1%) because of investigator withdrawal of study product. Mean participant adherence to study product was 98%, with 686 (86%) participants receiving every expected injection and 74 (9%) missing just one injection. Study product was discontinued entirely in eight participants who developed uncontrolled hypertension (four receiving 17P and four receiving placebo) and in one participant receiving 17P who developed generalised urticaria.

**Figure 2:**
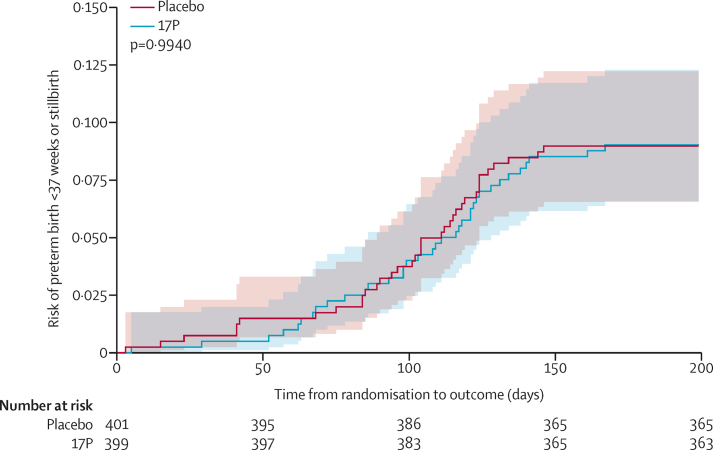
Incidence of preterm birth or stillbirth by treatment group 17P=17-alpha hydroxyprogesterone caproate.

The primary composite outcome of preterm birth or stillbirth was assessed in all 800 participants and occurred in 36 (9%) of 399 patients assigned to 17P and 36 (9%) of 401 patients assigned to placebo (risk difference [RD] 0·1%, 95% CI −3·9 to 4·0; RR 1·0, 95% CI 0·6 to 1·6; p=0·98, table 2). Preterm delivery of a liveborn infant occurred in 26 (7%) participants in the 17P group and 25 (6%) in the placebo group (0·3%, −3·1 to 3·7). Stillbirth occurred in ten (3%) of 399 patients in the 17P group and 11 (3%) of 401 patients in the placebo group (−0·2%, −2·5 to 2·0). The risk of the primary outcome as a function of time from random assignment was similar between treatment groups (figure 2).

**Table 2:** Primary and secondary outcomes by treatment assignment

		**17P (n=399)**	**Placebo (n=401)**	**Risk difference (95% CI)**	**Relative risk (95% CI)**	**Heterogeneity**
**Primary outcome**						
Delivery before 37 weeks of gestation or stillbirth at any gestational age (composite)		36 (9%)	36 (9%)	0·1% (−3·9 to 4·0)	1·0 (0·6 to 1·6)	p=0·98*
Livebirth before 37 weeks of gestation		26 (7%)	25 (6%)	0·3% (−3·1 to 3·7)	1·0 (0·6 to 1·8)	..
Stillbirth at any gestational age		10 (3%)	11 (3%)	−0·2% (−2·5 to 2·0)	0·9 (0·4 to 2·1)	..
**Secondary outcomes**						
Delivery before 37 weeks of gestation		31 (8%)	35 (9%)	−1·0% (−4·8 to 2·9)	0·9 (0·6 to 1·4)	..
	Spontaneous delivery before 37 weeks of gestation†	25/393 (6%)	26/392 (7%)	−0·3% (−3·7 to 3·2)	1·0 (0·6 to 1·6)	..
	Provider-initiated delivery before 37 weeks of gestation‡	6/374 (2%)	9/375 (2%)	−0·8% (−2·8 to 1·2)	0·7 (0·2 to 1·9)	..
Delivery before 34 weeks of gestation		14 (4%)	16 (4%)	−0·5% (−3·1 to 2·2)	0·9 (0·4 to 1·8)	..
Delivery before 28 weeks of gestation		3 (1%)	5 (1%)	..	..	..
**Subgroup analyses (primary composite outcome)**						
ART initiation						p=0·36§
	ART initiation in pregnancy	8/104 (8%)	12/112 (11%)	−3·0% (−10·7 to 4·7)	0·7 (0·3 to 1·7)	..
	ART initiation before conception	28/295 (9%)	24/289 (8%)	1·2% (−3·4 to 5·8)	1·1 (0·7 to 1·9)	..
Parity						p=0·66§
	Nulliparous	8/76 (11%)	7/81 (9%)	1·9% (−7·3 to 11·1)	1·2 (0·5 to 3·2)	..
	Parous	28/323 (9%)	29/320 (9%)	−0·4% (−4·8 to 4·0)	1·0 (0·6 to 1·6)	..
Estimated gestational age at random assignment						p=0·68§
	<20 weeks	18/228 (8%)	18/247 (7%)	0·6% (−4·2 to 5·4)	1·1 (0·6 to 2·0)	..
	≥20 weeks	18/171 (11%)	18/154 (12%)	−1·2% (−8·0 to 5·7)	0·9 (0·5 to 1·7)	..

Data are n (%) or n/N (%) unless otherwise specified. 17P=17 alpha-hydroxyprogesterone caproate. ART=antiretroviral therapy.

* χ^2^ test.

† 15 provider-initiated preterm births are excluded.

‡ 51 spontaneous preterm births are excluded.

§ Breslow-Day test for homogeneity. Statistical tests were not done when there were fewer than five events in either group.

The proportions of women who delivered spontaneously or with provider initiation before week 37 did not differ between treatment groups (table 2). Delivery for any indication before 28 gestational weeks and before 34 gestational weeks were also similar between treatment groups (table 2).

Other analyses showed no difference between groups in composite primary outcome (table 2). Among 216 participants who had initiated ART in pregnancy, the outcome was met by 8% of those assigned to 17P and 11% assigned to placebo. Among 584 women who initiated ART before pregnancy, 9% in the 17P group met the primary composite outcome compared with 8% in the placebo group. In the unplanned secondary analyses, risks of the primary composite outcome were similar in subgroups of nulliparous and parous participants and among women randomly assigned before or after 20 weeks of gestation (table 2).

Incidences of most maternal and neonatal adverse events were similar between treatment groups (table 3). Participants in the 17P group had a lower risk of delivering an infant with birthweight for gestational age in the 3rd percentile compared with those randomly assigned to placebo (RD −4·8%, 95% CI −8·9 to −0·7; RR 0·6, 95% CI 0·4 to 0·9). Conversely, liveborn infants born to mothers who received 17P had elevated risks of both 5-min Apgar score of less than 7 and neonatal death, although these comparisons were imprecise because events were few. Statistical tests were not done when there were fewer than five events in either group because statistical testing on these small numbers would not be meaningful (table 3). Adverse events deemed related to study product were reported by 140 (18%) of 800 participants and occurred in similar proportions in both randomisation groups (table 4). The most common adverse events were headache, diarrhoea, cough, itching, and rash. No serious adverse drug reactions were reported. Four patients in each group discontinued treatment due to uncontrolled hypertension not related to treatment and one patient assigned to 17P discontinued treatment due to generalised urticaria. Full details of all adverse events are reported in the appendix.

**Table 3:** Maternal and neonatal outcomes by treatment assignment

	**17P (n=399)**	**Placebo (n=401)**	**Risk difference (95% CI)**	**Relative risk (95% CI)**
**Maternal outcomes**				
Pregnancy-induced hypertension	13 (3%)	6 (2%)	1·8% (−0·3 to 3·9)	2·2 (0·8 to 5·7)
Pre-eclampsia	6 (2%)	9 (2%)	−0·7% (−2·6 to 1·1)	0·7 (0·2 to 1·9)
Eclampsia	0 (0%)	1 (<1%)	..	..
Maternal death	0 (0%)	1 (<1%)	..	..
Antepartum haemorrhage	6 (2%)	7 (2%)	−0·2% (−2·0 to 1·5)	0·9 (0·3 to 2·5)
Preterm prelabour rupture of membranes	5 (1%)	7 (2%)	−0·5% (−2·2 to 1·2)	0·7 (0·2 to 2·2)
Oligohydramnios	2 (1%)	2 (1%)	..	..
Polyhydramnios	22 (6%)	19 (5%)	0·8% (−2·3 to 3·8)	1·2 (0·6 to 2·1)
Chorioamnionitis	1 (<1%)	0 (0%)	..	..
Caesarean delivery	73 (18%)	77 (19%)	−0·9% (−6·3 to 4·5)	1·0 (0·7 to 1·3)
**Neonatal outcomes**				
Birthweight <10th percentile for gestational age*	95/392 (24%)	93/394 (24%)	0·6% (−5·3 to 6·6)	1·0 (0·8 to 1·3)
Birthweight <3rd percentile for gestational age*	28/392 (7%)	47/394 (12%)	−4·8% (−8·9 to −0·7)	0·6 (0·4 to 0·9)
Birthweight <2500 g†	41/395 (10%)	46/395 (12%)	−1·3% (−5·6 to 3·1)	0·9 (0·6 to 1·3)
Birthweight <1500 g†	7/395 (2%)	7/395 (2%)	0·0% (−1·8 to 1·8)	1·0 (0·4 to 2·8)
1-min Apgar score <7 among liveborn infants‡	12/336 (4%)	9/338 (3%)	0·9% (−1·7 to 3·5)	1·3 (0·6 to 3·1)
5-min Apgar score <7 among liveborn infants§	7/137 (5%)	2/144 (1%)	..	..
Neonatal intensive care unit admission among liveborn infants¶	29/389 (7%)	24/390 (6%)	1·3% (−2·2 to 4·8)	1·2 (0·7 to 2·0)
Neonatal supplemental oxygen requirement among liveborn infants¶	13/389 (3%)	10/390 (3%)	0·8% (−1·6 to 3·2)	1·3 (0·6 to 2·9)
Neonatal assisted ventilation among liveborn infants¶	1/389 (<1%)	1/390 (<1%)	..	..
Neonatal death‖	14/388 (4%)	7/386 (2%)	1·8% (−0·5 to 4·1)	2·0 (0·8 to 4·9)
Infant HIV infection**	1/374 (<1%)	1/379 (<1%)	..	..

Data are n (%) or n/N (%) unless otherwise specified. Statistical tests were not done when there were fewer than five events in either group. 17P=17 alpha-hydroxyprogesterone caproate.

* Birthweight not recorded for 10 neonates and birthweight centile not able to be calculated for another 4 neonates.

† Birthweight not recorded for 10 neonates.

‡ 21 stillborn infants excluded and 1-minute Apgar not recorded for 105 liveborn infants.

§ 21 stillborn infants excluded and 5-minute Apgar score not recorded for 498 liveborn infants.

¶ 21 stillborn infants excluded.

‖ 21 stillborn infants excluded and 5 infants with undocumented vital status at 28 days of life.

** 21 stillborn infants and 21 neonatal deaths excluded. 5 infants did not return for HIV testing at 6 weeks of life.

**Table 4:** Adverse drug events by treatment assignment

	**17P (n=399)**	**Placebo (n=401)**	**Risk difference (95% CI)**	**Relative risk (95% CI)**
Headache	25 (6%)	17 (4%)	2·0% (−1·1 to 5·1)	1·5 (0·8 to 2·7)
Diarrhoea	18 (5%)	10 (2%)	2·0% (−0·5 to 4·6)	1·8 (0·8 to 3·9)
Cough	14 (4%)	8 (2%)	1·5% (−0·8 to 3·8)	1·8 (0·7 to 4·1)
Itching	9 (2%)	12 (3%)	−0·7% (−3·0 to 1·5)	0·8 (0·3 to 1·8)
Rash	12 (3%)	5 (1%)	1·8% (−0·2 to 3·8)	2·4 (0·9 to 6·8)
Pain	5 (1%)	9 (2%)	−1·0% (−2·8 to 0·8)	0·6 (0·2 to 1·7)
Swelling	8 (2%)	6 (2%)	0·5% (−1·3 to 2·3)	1·3 (0·5 to 3·8)
Nausea or vomiting	4 (1%)	7 (2%)	..	..
Dizziness	0 (0%)	2 (<1%)	..	..
Urticaria	1 (<1%)	0 (0%)	..	..
Erythema	0 (0%)	1 (<1%)	..	..
Nodule	0 (0%)	0 (0%)	..	..
Anaphylaxis	0 (0%)	0 (0%)	..	..

Data are n (%) or n/N (%) unless otherwise specified. Statistical tests were not done when there were fewer than five events in either group. 17P=17 alpha-hydroxyprogesterone caproate.

## Discussion

In this trial, weekly injections of 17P initiated between 16 and 24 weeks of gestation did not reduce the risk of preterm delivery or stillbirth in women with HIV and no history of spontaneous preterm birth. We observed no effect on more severe definitions of prematurity, no effect among nulliparous women, and no effect among women who began weekly injections before 20 weeks of gestation. By contrast with some reports,^4^ women who started ART before pregnancy had a risk of preterm birth or stillbirth similar to that of women who initiated treatment during pregnancy, and no effect of 17P was observed in either of these subgroups. Adverse events, including drug-related reactions, were similar between randomisation groups, except for a reduced risk of infants very small for gestational age at birth in patients who received 17P.

The biological mechanisms that drive the elevated risk of adverse birth outcomes in women living with HIV have not been definitively established, but growing evidence implicates inflammation, immune activation, and altered placental angiogenesis.^15^, ^16^, ^17^ We hypothesised that HIV-related preterm birth and stillbirth might be ameliorated by the broad anti-inflammatory effects of progestogen supplementation,^17^, ^18^ but instead found a remarkably similar frequency of these outcomes between treatment groups. We also did not observe a benefit of 17P in a secondary analysis limited to preterm deliveries that occurred spontaneously (the phenotype that is frequently associated with inflammation^19^).

In a 2003 trial by Meis and colleagues in the USA,^20^ weekly 17P injections reduced the risk of recurrent preterm delivery, a finding supported by a subsequent meta-analysis that included additional smaller trials.^6^ Since then, 17P has been used routinely for this indication in North America and parts of Europe. However, the PROLONG trial^9^ (which was larger than Meis and colleagues' trial and of similar design but enrolled women at markedly lower risk of preterm delivery) found no benefit of 17P. In response to the findings of PROLONG, a mandated confirmatory trial, a US Food and Drug Administration advisory committee recommended approval of 17P be withdrawn for prevention of recurrent preterm birth.^21^ Despite this recommendation, both the American College of Obstetricians and Gynecologists and the Society for Maternal Fetal Medicine continue to support the use of 17P to prevent recurrent preterm birth, particularly among patients at very high risk in the USA.^22^, ^23^

The overall frequency of preterm birth or stillbirth in the current study was substantially lower than regional estimates^24^ and among the lowest ever reported among women living with HIV.^1^ One reason for this is the exclusion of women with previous spontaneous preterm delivery, and the resultant high proportion of parous women with previous term deliveries. The low overall risk of preterm birth and stillbirth in our trial might also be partly attributed to differences in HIV disease severity, immune suppression, or ART use compared with earlier reports. However, two contemporaneous cohorts drawn from the same patient population in Lusaka reported substantially higher rates of this composite outcome,^25^, ^26^ a finding that persisted even after applying the IPOP trial's inclusion and exclusion criteria (data not shown). One important way that the IPOP trial differs from these studies is in its weekly visit schedule. Each participant's appointments occurred on the same day each week, such that an inadvertent group care model arose in which women assembled and underwent group counselling with the same cohort throughout their pregnancy. Some trials of group-based antenatal care have reported improved perinatal outcomes in high-income settings,^27^ and increased antenatal attendance, facility delivery, and postnatal service use in sub-Saharan Africa.^28^

This trial supports the safety of 17P administration during pregnancy. Adverse drug reactions occurred equally between women randomly assigned to active and placebo study product, and overall incidence was consistent with previous trials of 17P in pregnancy.^9^, ^20^ The finding of a reduction in risk of small for gestational age among infants born to women randomly assigned to 17P warrants further investigation, particularly when taken in the context of findings from mouse models, in which progesterone improved ART-related placental vascular abnormalities.^17^ Finally, only two infants in the trial were diagnosed with HIV at the final 6-week visit (one in each randomisation group), which supports the efficacy of ART to prevent perinatal transmission and provides reassurance that 17P does not promote it.

We acknowledge several limitations of this trial. First, almost all our participants received tenofovir, emtricitabine, and efavirenz for treatment of their HIV infection. This combination remains the most common treatment prescribed to Zambian adults but is likely to be replaced over time by dolutegravir-containing regimens. The IMPAACT 2010 trial^29^ found a lower composite risk of early pregnancy loss, stillbirth, preterm delivery, or small for gestational age among women newly starting a combination of dolutegravir, tenofovir alafenamide fumarate, and efavirenz in pregnancy compared with those newly starting the regimen used in our trial (24·1% *vs* 32·7%, p=0·047). Therefore, our findings might not be generalisable if dolutegravir replaces efavirenz as first line treatment. Second, we designed this study to be relevant to our local context, where women present to care later in pregnancy. Thus, some women began the 17P intervention later in gestation than in other trials.^9^, ^20^ We note that restricting the analysis to women who were randomly assigned before 20 weeks did not affect our results (table 2). Third, our sample size was based on an event rate higher than observed, such that the estimated difference between treatment groups is less precise than planned. However, the risk of the primary outcome was similar in both groups, and the 95% CI suggests a difference larger than 4% is unlikely.

Administration of weekly 17P did not reduce the risk of preterm birth or stillbirth among Zambian women with HIV and no previous spontaneous preterm birth. A low risk of preterm birth in both randomisation groups could have resulted from other participant characteristics or trial procedures unrelated to the intervention.

## Data sharing

Deidentified individual participant data will be made available beginning 3 months and ending 5 years following Article publication for researchers who provide a methodologically sound proposal to achieve aims in the approved protocol. Proposals should be directed to the corresponding author, and data requestors will need to sign a data access agreement.

## Declaration of interests

We declare no competing interests.
