# Supplementary material for: Weekly 17 alpha-hydroxyprogesterone caproate to prevent preterm birth among women living with HIV: a randomised, double-blind, placebo-controlled trial
Source: Lancet HIV. 2021 Sep 9;8(10):e605–13. doi: 10.1016/S2352-3018(21)00150-8 (PMC8476342; doi:10.1016/S2352-3018(21)00150-8)
Supplement: Supplementary appendix [file mmc1.pdf]

THE LANCET
HIV

Supplementary appendix

This appendix formed part of the original submission and has been peer reviewed. We post it as supplied by the authors.

Supplement to: Price JT, Vwalika B, Freeman BL, et al. Weekly 17 alpha-hydroxyprogesterone caproate to prevent preterm birth among women living with HIV: a randomised, double-blind, placebo-controlled trial. *Lancet HIV* 2021; published online Sept 9. http://dx.doi.org/10.1016/S2352-3018(21)00150-8.

APPENDIX Table 1. Adverse events by treatment assignment in the IPOP trial, February 2018–August 2020

	**17P N = 399**		**Placebo N = 401**	
**Adverse event**	**n**	**%**	**n**	**%**
ANEMIA	170	43	200	50
URINARY TRACT INFECTION	81	20	78	19
SYPHILIS	60	15	61	15
HEADACHE	53	13	41	10
LOWER ABDOMINAL OR PELVIC PAIN	48	12	41	10
DIARRHEA	29	7	26	6
VOMITING	22	6	22	5
COUGH	21	5	15	4
COMMON COLD / FLU	18	5	11	3
RASH	15	4	7	2
VULVOVAGINAL ITCHING	14	4	22	5
ABDOMINAL PAIN	13	3	14	3
EDEMA OF LOWER EXTREMITIES	13	3	8	2
BACKACHE	12	3	24	6
VULVOVAGINAL WARTS OR SORES	10	3	7	2
DIZZINESS	8	2	9	2
VAGINAL BLEEDING	8	2	9	2
NAUSEA AND VOMITING	7	2	10	2
PAIN OF THE LOWER LIMBS	7	2	6	1
SWELLING ON INJECTION SITE	7	2	7	2
DYSURIA	5	1	3	1
FEVER	5	1	5	1
ITCHING	5	1	1	0
PAIN AT INJECTION SITE	5	1	6	1
VAGINAL DISCHARGE	5	1	9	2
ITCHING AT INJECTION SITE	4	1	13	3
JOINT PAIN	4	1	0	0
UPPER RESPIRATORY INFECTION	4	1	1	0
VAGINAL CANDIDIASIS	4	1	6	1
POSTPARTUM HEMORRHAGE	3	1	3	1
UPPER ABDOMINAL PAINS	3	1	0	0
BAD SMELLING VAGINAL ODOR	2	1	1	0
CHEST PAIN	2	1	4	1
CONSTIPATION	2	1	0	0
FATIGUE	2	1	4	1
HEMORRHOIDS	2	1	4	1

INTRAUTERINE GROWTH RESTRICTION	2	1	5	1
MALARIA	2	1	2	0
NAUSEA	2	1	2	0
PERINEAL TEAR	2	1	0	0
PILES	2	1	1	0
SIDE PAIN	2	1	2	0
SORE THROAT	2	1	1	0
TOOTHACHE	2	1	4	1
UMBILICAL PAIN	2	1	0	0
ASTHMA	1	0	1	0
BACTERIAL VAGINOSIS	1	0	0	0
BODY WEAKNESS	1	0	0	0
BORDERLINE CLINICAL DEPRESSION	1	0	0	0
CERVICAL LACERATION	1	0	0	0
CHANGE IN VISION	1	0	0	0
CRACKED NIPPLES	1	0	0	0
DOMESTIC ABUSE	1	0	0	0
DROWSINESS	1	0	0	0
DYSPNEA	1	0	0	0
EPILEPSY	1	0	0	0
EPISTAXIS	1	0	3	1
EYE INJURY	1	0	0	0
FRACTURE OF LEFT ANKLE	1	0	0	0
GASTROENTERITIS	1	0	1	0
GINGIVITIS	1	0	0	0
HEAT AFTER INTERCOURSE	1	0	0	0
HEMOPERITONEUM	1	0	0	0
HYDRONEPHROSIS	1	0	0	0
HYPEREMESIS GRAVIDARUM	1	0	0	0
HYPOTENSION	1	0	0	0
INFLAMED TONSILS	1	0	0	0
KAPOSI'S SARCOMA	1	0	0	0
LEFT EYE FACIAL LACERATIONS	1	0	0	0
LEFT NOSTRIL LACERATION	1	0	0	0
LYMPHADENOPATHY	1	0	1	0
NEONATAL SEPSIS	1	0	1	0
NUMBNESS OF LOWER LIMBS	1	0	0	0
NUMBNESS OF UPPER LIMBS	1	0	0	0
ORAL THRUSH	1	0	0	0
PAIN UNDER THE BREAST	1	0	0	0
PERFORATED SMALL INTESTINES	1	0	0	0

PLEURAL EFFUSION	1	0	0	0
PRESUMED PSYCHOSIS	1	0	0	0
PUERPERAL SEPSIS	1	0	0	0
PYELONEPHRITIS	1	0	0	0
RINGWORM	1	0	0	0
SCAR TENDERNESS	1	0	0	0
SNEEZING	1	0	1	0
SORES ON THE ANUS	1	0	0	0
SWELLING AROUND THE EYES	1	0	0	0
SWELLING OF THE JAW	1	0	0	0
SWELLING OF THE UPPER LIP	1	0	0	0
SWELLING ON THE LEFT BREAST	1	0	0	0
SWOLLEN LABIA	1	0	0	0
URTICARIA	1	0	0	0
ABSCESS ON THE FOREHEAD	0	0	1	0
ACNE	0	0	1	0
BREAST LUMP	0	0	1	0
CERVICITIS	0	0	1	0
DIABETES MELLITUS	0	0	1	0
EDEMA OF UPPER EXTREMITIES	0	0	1	0
ERYTHEMA AT INJECTION SITE	0	0	1	0
GENERAL BODY MALAISE	0	0	1	0
GESTATIONAL DIABETES	0	0	1	0
HEART PALPITATIONS	0	0	2	0
HERPES ZOSTER	0	0	2	0
HYSTERIA	0	0	1	0
LEFT ADNEXAL CYST	0	0	1	0
LOSS OF APPETITE	0	0	1	0
LOSS OF WEIGHT	0	0	1	0
LOW-LYING PLACENTA	0	0	1	0
PAIN OF INCISION	0	0	1	0
PRESUMED PNEUMONIA	0	0	1	0
PROTEINURIA	0	0	1	0
PULMONARY TUBERCULOSIS	0	0	2	0
SWEATING	0	0	1	0
SWELLING ON LABIA	0	0	1	0
SYNCOPE	0	0	2	0
URINARY BLADDER INJURY	0	0	1	0
VARICOSE VEINS	0	0	1	0

Data are n (%). 17P=17 alpha-hydroxyprogesterone caproate
